# ApoE4 as a Therapeutic Target in Epilepsy: From Pathogenic Mechanisms to Precision Medicine

**DOI:** 10.1002/cns.71167

**Published:** 2026-09-22

**Authors:** Kang Pan, Keyi Zhu, Yajun E, Yucang He, Siqi Ding

**Affiliations:** ^1^ Wenzhou Medical University Wenzhou Zhejiang Province People's Republic of China; ^2^ The Affiliated Yiwu Hospital of Wenzhou Medical University Yiwu City Zhejiang Province People's Republic of China; ^3^ First Affiliated Hospital of Wenzhou Medical University Wenzhou City Zhejiang Province People's Republic of China

**Keywords:** apolipoprotein E4, epilepsy, neuroinflammation, precision medicine

## Abstract

**Background:**

Epilepsy is a heterogeneous central nervous system disorder characterized by recurrent seizures and is frequently accompanied by cognitive and neuropsychiatric comorbidities. Although apolipoprotein E4 (ApoE4) is well established as a genetic risk factor in Alzheimer's disease, its role as a modifier of epilepsy susceptibility, progression, and therapeutic response remains incompletely understood.

**Objective:**

This review aims to summarize current evidence regarding the molecular mechanisms, clinical implications, and therapeutic potential of ApoE4 in epilepsy, with emphasis on its relevance to precision medicine.

**Methods:**

A narrative review was conducted by synthesizing preclinical and clinical studies investigating the relationship between ApoE4 and epilepsy, including mechanisms related to neuroinflammation, synaptic dysfunction, lipid metabolism, blood–brain barrier (BBB) integrity, clinical phenotypes, and targeted therapeutic strategies.

**Results:**

ApoE4 contributes to epileptogenesis through multiple interconnected pathways. It promotes neuroinflammatory responses by activating microglia and inflammatory signaling, disrupts synaptic plasticity through N‐methyl‐D‐aspartate receptor dysfunction and excitation‐inhibition imbalance, impairs lipid and energy metabolism leading to neuronal hyperexcitability, and compromises BBB integrity. Clinically, ApoE4 carriers show increased seizure susceptibility, accelerated cognitive decline, hippocampal atrophy, and variable responsiveness to antiseizure medications. Emerging therapeutic approaches include ApoE4 structural correction, modulation of metabolic and inflammatory pathways, and gene‐based interventions.

## Graphical abstract

ApoE4 acts as a key disease modifier in epilepsy by driving neuroinflammation, synaptic dysfunction, metabolic imbalance, and BBB disruption. These interacted mechanisms promote network hyperexcitability and disease progression. Targeting ApoE4 and its downstream pathways offers promising opportunities for precision therapy.

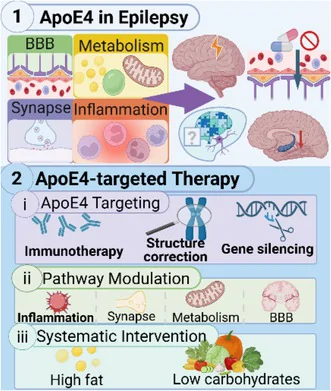

**Abbreviations**
ABCA1: ATP‐binding cassette sub‐family A member 1
ad
: Alzheimer's disease
AEP: asparagine endopeptidase
ApoE: apolipoprotein E
APP: amyloid precursor protein
ASMs: antiseizure medication
ASO: antisense oligonucleotide
Atg12: autophagy related 12
BBB: blood–brain barrier
C/EBPβ: CCAAT/enhancer binding protein beta
CNS: central nervous system
CSF: cerebrospinal fluid
DOPEGAL: 3,4dihydroxyphenylglycolaldehyde
E/I: excitation/inhibition
FoxO3a: forkhead box protein O3‐A
GSK‐3β: glycogen synthase kinase 3β
HDAC: histone deacetylase
IL‐1β: interleukin‐1β
NMDAR: N‐methyl‐D‐aspartate receptor
TAGLN3: transgelin 3
tau: microtubule‐associated protein tau
TLE: temporal lobe epilepsy
TTC39B: tetratricopeptide repeat domain 39B
VMAT2: vesicular monoamine transporter 2

## 1 Introduction

Epilepsy is a common yet highly heterogeneous central nervous system disorder. Its development and progression involve multiple interacting mechanisms, including abnormal neuronal excitability, neuroinflammation, and the disruption of metabolic homeostasis [1]. Beyond these canonical pathways, growing attention has focused on genetic and molecular modifiers that influence disease susceptibility and clinical heterogeneity. In this context, recent studies have increasingly examined the role of the lipid transporter apolipoprotein E (ApoE) in epilepsy.

APOE is a key lipid transporter in the central nervous system and is encoded by the APOE gene. Its major alleles include ε2, ε3, and ε4 [2]. Among them, the ApoE4 isoform exhibits reduced structural stability due to specific amino acid substitutions, leading to impaired lipid binding and decreased receptor affinity. Consequently, these alterations compromise lipid metabolic coupling between neurons and glial cells, membrane repair, and synaptic maintenance [2, 3, 4]. In addition to its role in lipid transport, ApoE4 activates pathogenic signaling pathways, including glycogen synthase kinase 3β (GSK‐3β) and asparagine endopeptidase (AEP), thereby promoting microtubule‐associated protein tau (tau) hyperphosphorylation and neuronal cell death [5, 6, 7]. Notably, these molecular effects converge on core pathological features of epilepsy, including neuroinflammation, synaptic dysfunction, and metabolic imbalance. Consistent with these mechanistic insights, clinical studies suggest that ApoE4 carriers exhibit a higher frequency of seizures [8], an increased prevalence of drug resistance [9], and accelerated hippocampal atrophy on neuroimaging [10]. Collectively, these findings underscore the pathogenic relevance of ApoE4 in epilepsy and support its potential utility as both a biomarker and a therapeutic target.

However, despite its well‐established role in neurodegenerative disorders, particularly Alzheimer's disease (AD), the involvement of ApoE4 in epilepsy remains comparatively underexplored. Significant knowledge gaps remain regarding its epilepsy‐specific molecular mechanisms, clinical implications, and translational therapeutic potential. To address these knowledge gaps, this review systematically evaluates the mechanistic actions, clinical impact, and potential targeted therapeutic strategies involving ApoE4 in epilepsy, to establish a conceptual framework for future mechanistic studies and individualized therapeutic approaches.

### 1.1 Search Strategy and Selection Criteria

The literature published from 1996 to August 1, 2025, was comprehensively searched in PubMed using the following terms in the title, abstract, or descriptors: APOE, apolipoprotein E, epilepsy, seizure, blood–brain barrier (BBB), neuroinflammation, tau, amyloid, GSK‐3β, and AD. The search focused on both preclinical studies (including animal models) and human studies investigating the relationship between ApoE4 and epilepsy, including molecular mechanisms, clinical phenotypes, cognitive outcomes, and therapeutic implications. Studies considered in this narrative review were peer‐reviewed original research articles published in English. The identified literature was qualitatively synthesized to integrate mechanistic insights and clinical evidence regarding ApoE4‐associated epileptic pathophysiology and potential therapeutic strategies.

## 2 Potential Molecular Mechanisms of ApoE4 in Epilepsy

**FIGURE 1:**
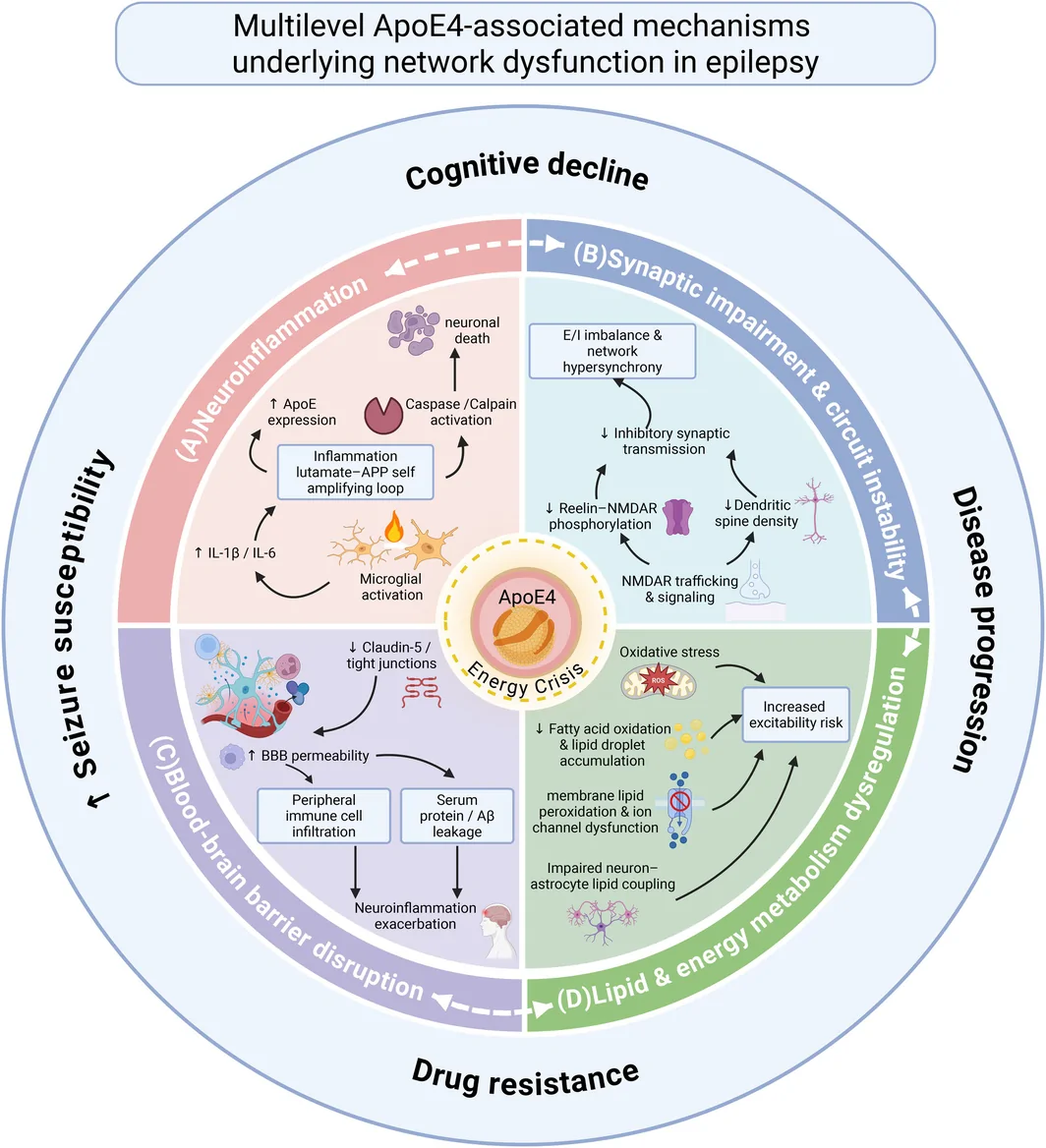
Multimodal mechanisms of ApoE4 in epilepsy. ApoE4 drives four interconnected pathological processes: (A) neuroinflammation via microglial activation and cytokine release; (B) synaptic dysfunction through NMDAR impairment and E/I imbalance; (C) BBB disruption with tight junction loss; and (D) metabolic dysregulation causing energy crisis. Dashed arrows indicate vicious cycles between modules. ApoE4, apolipoprotein E4; BBB, blood–brain barrier; E/I, excitation/inhibition; NMDAR, N‐methyl‐D‐aspartate receptor. Created with http://biorender.com/.

An increasing body of evidence indicates that ApoE4 may contribute to the onset and progression of epilepsy through multiple interrelated pathological pathways. These include the following: (i) amplification of neuroinflammatory responses, (ii) impairment of synaptic plasticity and destabilization of neural circuit activity, (iii) disruption of lipid and energy metabolism with consequent alterations in neuronal excitability, and (iv) compromise of BBB integrity. These mechanisms do not act independently. Instead, they interact with each other to promote network hyperexcitability and structural damage. Consequently, ApoE4 may increase seizure susceptibility and contribute to treatment resistance (Figure 1).

### 2.1 Exacerbation of Neuroinflammatory Responses

Much of the evidence linking ApoE4 to neuroinflammatory amplification currently derives from AD models and systemic inflammation paradigms; direct validation in isolated epilepsy models remains incomplete.

Neuroinflammation represents a critical pathological substrate of epilepsy, contributing to seizure frequency, severity, and the emergence of pharmacoresistance [11]. ApoE4 exacerbates neuroinflammation in epilepsy through multiple mechanisms. Specifically, ApoE4 promotes the polarization of microglia toward a pro‐inflammatory phenotype. It also reduces microglial phagocytic activity and impairs the clearance of neurotoxic molecules, including β‐amyloid and amyloid precursor protein (APP). These changes amplify downstream inflammatory signaling [12].

In diverse models of central inflammation, ApoE4 carrier status has been demonstrated to markedly increase the expression of pro‐inflammatory mediators, including IL‐1β and IL‐6 [13], which in turn drive immune cell infiltration and glial activation. Notably, a self‐amplifying “inflammation‐glutamate‐APP” circuit has been proposed [14]. In animal experiments, IL‐1β enhances glutamate release from neurons and upregulates the expression of APOE and βAPP, while soluble APP, Aβ, and glutamate further stimulate ApoE production. It should be noted, however, that while the disruption of β‐amyloid clearance and the “inflammation‐glutamate‐APP” circuit have been extensively documented in AD models, their direct causal contribution to spontaneous chronic seizures in isolated epilepsy models requires more rigorous validation.

Although this loop may exert transient compensatory effects in the early‐stage disease, its chronic activation results in persistent glutamate spillover, neuronal stress, and progressive neurodegeneration, thereby predisposing individuals to epilepsy and AD‐related pathology. Concurrently, some studies have described a metabolization‐inflammation‐cell death signaling pathway driven by ApoE4. The unique ketone body metabolic profile associated with ApoE4 may precipitate energy crises in hypoxic and ischemic environments. This leads to oxidative stress, subsequent activation of inflammatory cascades, and activation of caspase‐ and calpain‐dependent pathways. These events result in neuronal apoptosis and necrosis, and ultimately epilepsy [15].

Collectively, these findings suggest that ApoE4 not only amplifies seizure‐associated inflammation through dysregulated signaling loops but also functionally links epileptogenesis with neurodegenerative processes. This ApoE4‐driven signaling pathway directly damages neurons through metabolic disruption, inflammation, and cell death. It also creates a permissive environment for synaptic dysfunction and network hyperexcitability by impairing energy supply and membrane stability. Consequently, metabolic dysregulation serves as a central integrative hub linking inflammation to epilepsy susceptibility.

### 2.2 Synaptic Impairment and Circuit Instability

The core pathological substrate of epilepsy is balance disruption between excitation and inhibition within neural networks, with abnormalities in synaptic structure and function serving as central mediators [16]. ApoE4 has been demonstrated to induce synaptic dysfunction, encompassing alterations in synaptic transmission, plasticity, and morphology [17].

Mechanistically, ApoE4 selectively impairs APOE receptor trafficking and downstream signaling by interfering with N‐methyl‐D‐aspartate receptor (NMDAR)‐dependent pathways in transgenic AD mouse models, reduces receptor surface expression, and attenuates Reelin‐mediated NMDAR phosphorylation, thereby weakening glutamatergic receptor function and synaptic plasticity. Notably, functional deficits are evident even in early stages; young ApoE4 AD animals exhibit attenuated cortical neuronal activity and impaired plasticity prior to the onset of neurodegenerative pathology. These findings, spanning both early functional impairments and late‐stage Aβ pathological mechanisms in AD models, suggest a compromised neural environment that may disrupt inhibitory network regulation and predispose the brain to the hypersynchronous neuronal firing characteristic of epilepsy [18].

In addition, ApoE4 decreases dendritic spine density and stability, undermining both the formation of new synapses and the maintenance of existing ones [19]. Electrophysiological studies further demonstrate that neurons in ApoE4 carriers exhibit diminished responsiveness to inhibitory postsynaptic currents, rendering them intrinsically more susceptible to hyperexcitability.

Collectively, synaptic dysfunction and structural remodeling driven by ApoE4 destabilize neuronal circuits, thereby establishing a network‐level substrate for epileptogenesis. Notably, the maintenance of synaptic integrity is highly dependent on lipid and energy homeostasis, both of which are dysregulated in the presence of ApoE4, suggesting that its synaptic toxicity may be partly secondary to impaired metabolic substrate availability.

### 2.3 Metabolic Dysregulation and Neuronal Hyperexcitability

APOE is a central regulator of cerebral cholesterol and lipid metabolism. The ApoE4 isoform, with its reduced lipid‐binding and transport capacity, profoundly disrupts lipid homeostasis and thereby alters neuronal excitability, contributing to epileptogenesis. Accumulating evidence demonstrates that ApoE4 impairs brain lipid and energy metabolism by disturbing neuron‐astrocyte metabolic coupling, perturbing fatty acid metabolism, and compromising neuronal energy supply and function [6].

Extrapolated evidence from AD models and cellular systems suggests that ApoE4 disrupts lipid homeostasis in ways that may heighten neuronal excitability. Specifically, ApoE4 has been demonstrated to reduce fatty acid oxidation, promote intracellular lipid accumulation, and impair membrane integrity and ion channel activity, collectively heightening neuronal excitability [20, 21]. Besides, lipid metabolic dysregulation indirectly modulates neuronal excitability by altering neurotransmitter synthesis and release. Aberrant cholesterol metabolism can impair synaptic vesicle formation and trafficking, disturb neurotransmitter release, and induce an excitatory‐inhibitory imbalance, thereby increasing the seizure risk [22].

In patients with epilepsy, instability in lipid binding, distribution, and cholesterol homeostasis is associated with ApoE4, which further impairs membrane repair and plasticity, destabilizing neuronal membranes and predisposing to more frequent and pharmacoresistant seizures [23]. Moreover, the ApoE4 allele increases the risk of epilepsy following hypoxic–ischemic brain injury by disrupting glucose and ketone body metabolism and exacerbating inflammation and cell death [15].

Notably, metabolic dysregulation not only directly enhances neuronal excitability but also indirectly compromises BBB integrity by inducing oxidative stress and membrane lipid peroxidation, thereby reinforcing neuroinflammatory cascades and amplifying epilepsy susceptibility.

### 2.4 Compromise of BBB Integrity

Beyond its established role in AD‐related cerebrovascular pathology, ApoE4 has been implicated in BBB dysfunction, which permits peripheral inflammatory mediators and toxic molecules to infiltrate the brain parenchyma and represents a critical contributing factor to epileptogenesis [24]. ApoE4 has also been implicated in cerebrovascular pathology, promoting increased BBB permeability [25], and concurrent upregulation of oxidative stress pathways [26].

Under epileptogenic conditions, barrier disruption facilitates the entry of Aβ, serum proteins, and immune cells into neural tissue, thereby triggering neuroinflammation and modulating neuronal excitability [27]. Mechanistically, ApoE4 downregulates tight junction proteins such as claudin‐5 in AD models, weakening endothelial integrity and compromising barrier function [25].

Damage to BBB not only exposes neurons to harmful circulating molecules but also amplifies neuroinflammation and metabolic dysregulation, thereby establishing a self‐perpetuating pathophysiological cycle that exacerbates seizure susceptibility. Collectively, these multilevel, interconnected mechanisms provide a unifying mechanistic framework through which ApoE4 promotes epilepsy and provide a biological basis for its associated clinical manifestations, including higher seizure frequency, cognitive decline, and drug resistance. A structured summary of these mechanisms, associated molecules, and the levels of evidence is presented in Table 1.

**TABLE 1:** Molecular mechanisms and strength of evidence for ApoE4‐related epilepsy.

Mechanism link	Involved cells/molecules	Main pathological effects	Existing evidence types	Strength of direct evidence for epilepsy
Neuroinflammation	Microglia (IL‐1β↑), astrocytes	Promote excitotoxicity	Animal (Epilepsy and AD models) + clinical brain tissue	High
Synaptic plasticity disorder	NMDAR/Reelin reduction	Inhibition of synaptic transmission	Cells/AD transgenic mice	Medium
Lipid metabolism disorder	Cholesterol transport↓, membrane fluidity↓	Neuron excitability ↑	AD‐derived cell and animal models	Medium to low
BBB impairment	Claudin‐5 reduce, permeability↑	Pro‐inflammatory substances enter the brain	Animal/clinical brain imaging	Medium
Metabolism‐inflammation‐death pathway	Ketone Body Metabolizing Enzymes, Caspase/Calpain, Hypoxic/Ischemic Environment	ApoE4 uses ketone bodies to generate energy crisis during ischemia, causing neuron apoptosis	Animal (neonatal hypoxic–ischemic seizure models and AD models)	Medium
Glutaminergic mechanism of excitation‐inhibition imbalance	Presynaptic glutamate release	Glutamate overflow; impaired NMDAR function, increased network excitability.	Animal	Medium

*Note:* “Involved cells/molecules” lists the key effectors examined in the cited studies. “Main pathological effects” highlights the functional consequence most relevant to seizure generation or propagation. “Existing evidence types” indicates whether supporting data derive from cell culture, animal models, human brain tissue, or clinical imaging. “Strength of direct evidence for epilepsy” is a semi‐quantitative grading (high/medium/low) based on the number of independent studies, replication across species, and specificity for epilepsy versus other neurodegenerative conditions.

Abbreviations: BBB, blood–brain barrier; IL‐1β, interleukin‐1β; NMDAR, N‐methyl‐D‐aspartate receptor; ROS, reactive oxygen species.

## 3 Prognostic Implications of ApoE4 in Epilepsy

**FIGURE 2:**
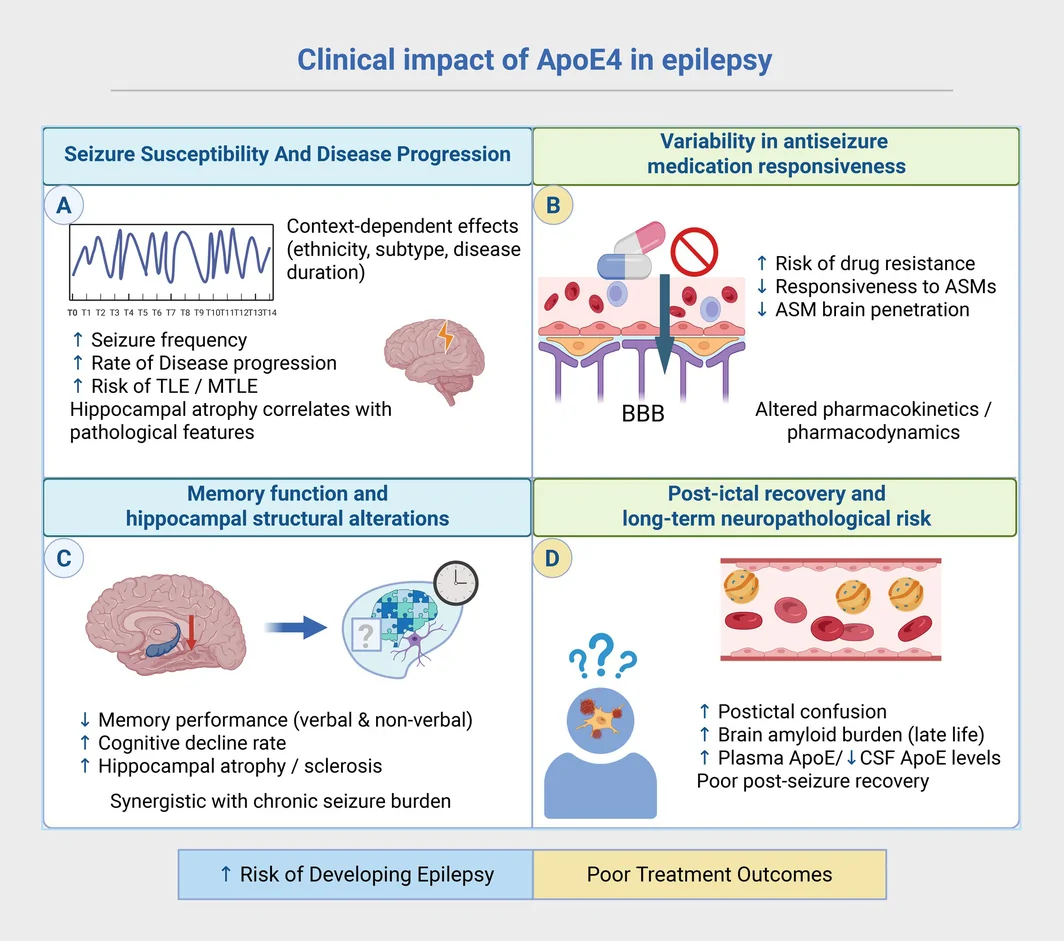
Clinical impact of ApoE4 in epilepsy. Four quadrants illustrate ApoE4‐associated clinical phenotypes: (A) increased seizure susceptibility and disease progression; (B) drug resistance and altered pharmacokinetics; (C) accelerated cognitive decline and hippocampal atrophy; and (D) elevated postictal confusion and amyloid burden. Arrows indicate upregulation (↑) or downregulation (↓). ApoE4, apolipoprotein E4; ASMs, antiseizure medications; BBB, blood–brain barrier; MTLE, mesial temporal lobe epilepsy; TLE, temporal lobe epilepsy. Created with http://biorender.com/.

Accumulating clinical and epidemiological evidence indicates that ApoE4 functions not only as a genetic susceptibility factor but also as a disease‐modifying determinant that significantly influences seizure burden, disease progression, cognitive outcomes, and therapeutic responsiveness in epilepsy. Collectively, these effects suggest that ApoE4 fundamentally influences the natural history and clinical heterogeneity of epilepsy (Figure 2).

### 3.1 Seizure Susceptibility and Disease Progression

Multiple clinical and epidemiological studies have demonstrated that carriers of the ApoE4 allele are at increased risk of seizures and often experience a more unfavorable disease trajectory. In several epilepsy cohort studies, ApoE4 carriers exhibited higher seizure frequencies and more rapid disease progression compared with non‐carriers [28, 29]. Cohort studies, case–control studies, and meta‐analyses further demonstrate a robust association between ApoE4 and medial temporal lobe epilepsy (MTLE). In multiple genetic models (e.g., ε3/ε4 versus ε3/ε3), APOE ε4 carriers demonstrated a significantly increased risk of TLE/MTLE [30], alongside longer disease duration, and more severe cognitive impairment [31]. These associations appear particularly robust in Chinese populations and well‐characterized population‐based studies (carrier ε4 versus ε3 model: Odds ratio [OR] = 1.51, 95% confidence interval [CI] = 1.05–2.18; *p* = 0.028) [32]. Notably, previous studies have failed to detect a significant association between APOE ε4 and TLE risk or age of onset [33], thereby emphasizing the context‐dependent and heterogeneous nature of ApoE4 effects. It is widely hypothesized that ApoE4 may exacerbate epilepsy‐related cognitive impairment by impairing neuronal repair mechanisms or promoting neurodegeneration.

In terms of structural imaging, ApoE4 carriers with specific types of epilepsy, especially temporal lobe epilepsy, often present with distinct neuroanatomical abnormalities, including hippocampal atrophy [10], and/or hippocampal sclerosis [34]. These progressive structural changes closely correlate with seizure frequency, pharmacoresistance, and cognitive decline, suggesting that ApoE4 contributes to epilepsy progression by accelerating neurodegenerative processes within seizure‐prone neural networks.

### 3.2 Memory Function and Hippocampal Structural Alterations

Beyond seizure burden, ApoE4 is strongly associated with adverse cognitive outcomes in epilepsy, particularly in memory‐related domains. Among elderly individuals, seizures in ApoE4 carriers are frequently accompanied by accelerated rates of cognitive decline [19]. Compared with non‐carriers, epilepsy patients carrying ApoE4 demonstrate more rapid and pronounced deterioration in cognitive performance, a pattern that is also observed following the onset of new seizures.

Importantly, evidence suggests a robust and synergistic interaction between chronic seizures and ApoE4, whereby dual exposure leads to rates of cognitive decline that substantially exceed the additive effects of either factor alone. Studies have demonstrated a significant interaction between APOE ε4 status and epilepsy duration: ApoE4 carriers with long‐standing epilepsy perform worst in verbal and non‐verbal memory tests, a deficit that persists before and after temporal lobectomy [31].

These findings indicate that ApoE4 amplifies the long‐term cognitive burden associated with epilepsy, likely through cumulative hippocampal damage and impaired neural repair. From a clinical perspective, this interaction underscores the need to consider genetic background and disease duration when assessing cognitive prognosis and surgical outcomes. Nevertheless, current evidence is derived from relatively small cohorts and specific epilepsy subtypes, thereby underscoring the need for large‐scale, longitudinal studies across diverse epilepsy syndromes.

### 3.3 Variability in Antiseizure Medication Responsiveness

ApoE4 has also been implicated in interindividual variability in antiseizure medication (ASM) responsiveness, representing a potential contributor to treatment resistance [35]. Despite ASMs remaining the cornerstone of epilepsy management, approximately 30% of patients develop drug‐resistant epilepsy [36]. Emerging evidence suggests that the APOE genotype influences pharmacokinetic and pharmacodynamic processes, including drug absorption, distribution, metabolism, and central nervous system penetration [35]. Specifically, ApoE4 carriers have been reported to exhibit reduced responsiveness to certain ASMs [9].

Mechanistically, ApoE4 may influence drug transporter expression and function, thereby limiting effective BBB permeability to therapeutic agents and reducing ASM concentrations within neural tissue. Concurrently, ApoE4 may alter neuronal sensitivity to ASMs by modulating neurotransmitter systems and intracellular signaling pathways, further compromising therapeutic efficacy.

Collectively, these observations underscore the role of ApoE4 as a pharmacogenetic modifier of treatment response in epilepsy and highlight the need for precision medicine‐driven therapeutic strategies to optimize therapy in genetically susceptible populations.

### 3.4 Post‐Ictal Recovery and Long‐Term Neuropathological Risk

In addition to influencing seizures and treatment outcomes, ApoE4 modulates broader clinical manifestations of epilepsy, including post‐ictal recovery and long‐term neuropathological risk. Studies have reported elevated plasma ApoE levels in patients with epilepsy [37], while case–control studies demonstrate reduced cerebrospinal fluid ApoE concentrations during seizures [38]. These changes reflect dynamic, seizure‐induced alterations rather than stable genotypic differences, possibly linked to acute neural injury and repair demands.

Clinically, a retrospective study of patients with drug‐refractory TLE revealed that ApoE4 carriers exhibited a significantly higher incidence of postictal recovery compared with non‐carriers (OR = 2.86, 95% CI = 1.02–7.97; *p* = 0.045) [39], suggesting impaired post‐seizure recovery mechanisms. Moreover, cross‐sectional studies indicate that individuals with childhood epilepsy, particularly ApoE4 carriers, display higher cerebral amyloid burden in later life [40].

Collectively, these observations suggest that ApoE4 confers increased vulnerability not only to acute post‐seizure dysfunction but also to long‐term neurodegenerative sequelae, thereby linking epilepsy to broader trajectories of brain aging. Dynamic changes in ApoE protein levels may thus serve as biomarkers of neural stress and repair capacity, underscoring the importance of long‐term monitoring and early intervention in genetically susceptible epilepsy populations. Furthermore, we present a comprehensive and structured synthesis of clinical studies examining the relationship between ApoE4 carrier status and epileptic phenotypes, including study design, key findings, effect sizes, and corresponding references (Table 2).

**TABLE 2:** Correlation between ApoE4 and clinical characteristics of epilepsy.

Clinical features	Research type/race	Main findings	Key Indicators/Results	References
Epilepsy susceptibility and disease progression	Cohort studies, case–control studies, meta‐analyses	ApoE4 carriers have more frequent seizures and faster disease progression	ε4 vs. ε3:OR = 1.51, 95% CI = 1.05–2.18, *p* = 0.028	[28, 29, 30, 31]
Structural image features	TLE patients	Hippocampal sclerosis more common in ApoE4 carriers	It is dose‐dependent (ε4/ε4 > ε3/ε4 > no ε4)	[34]
Epilepsy type association	Multiple genetic models (e.g., ε3ε4 vs. ε3ε3 or ε4ε4 vs. ε3ε4)	ApoE4 is associated with TLE/MTLE	ApoE4 is associated with longer course of disease and more severe cognitive impairment in TLE	[30, 31]
Cognitive function and memory impairment	TLE patients	ApoE4 is associated with accelerated cognitive decline in epilepsy patients	The cognitive function scores and memory scores of TLE patients who carry APOE ε4 are lower than those of non‐carriers.	[30, 31]
Differences in drug response	AD patients	ApoE4 carriers have poorer response to Antiseizure Medications (ASMs)	Affects lipid transport, Aβ metabolism, Tau/inflammation, cerebrovascularity, epigenetics	[9]
Recovery after attack	Retrospective study (TLE patients)	ApoE4 carriers have higher incidence of postictal confusion	OR = 2.86, 95% CI = 1.02–7.97, *p* = 0.045	[39]
Changes in ApoE protein levels	Case control study	Plasma ApoE is increased and cerebrospinal fluid ApoE is decreased in patients with epilepsy	CSF‐ApoE decrease may be a post‐seizure reaction.	[37, 38]

*Note:* Clinical studies published between 1997 and 2025 (*n* = 13) were integrated to evaluate the modifying role of ApoE4 on epilepsy phenotype. Research type includes prospective cohort, retrospective case–control, cross‐sectional imaging, and population‐based meta‐analyses; race/ethnicity is specified when > 80% of the sample derived from a single ancestry. “Key indicators/results” present the primary quantitative outcome (odds ratio, standardized mean difference, or radiological score) with 95% confidence intervals and exact *p*‐values when available.

Abbreviations: ASM, antiseizure medication; CI, confidence interval; CSF, cerebrospinal fluid; MTLE, mesial temporal lobe epilepsy; OR, odds ratio; TLE, temporal lobe epilepsy.

However, several translational challenges must be addressed before dynamic ApoE alterations can be implemented as clinically actionable post‐ictal biomarkers. A primary challenge lies in defining the optimal temporal window for post‐ictal sample collection. Seizure‐induced neural stress triggers rapid, time‐sensitive proteomic shifts. Therefore, immediate serial monitoring is required to capture peak alterations before baseline re‐equilibration occurs [38]. However, obtaining immediate post‐ictal cerebrospinal fluid (CSF) via lumbar puncture presents profound ethical and practical hurdles in emergency or neuro‐intensive care settings, especially during post‐ictal confusion or status epilepticus.

Furthermore, as detailed in Section 2.4, acute seizure activity induces profound, spatially heterogeneous blood–brain barrier (BBB) disruption [41]. This barrier leakage severely confounds the interpretation of plasma‐to‐CSF ApoE concentration ratios, as peripheral ApoE influx or central protein extravasation masks authentic central nervous system repair dynamics [37]. To transform these dynamic fluctuations into a viable point‐of‐care assay for neural stress, future translational efforts must focus on: (1) establishing high‐sensitivity, non‐invasive blood or salivary assays capable of isolating central CNS‐derived exosomal ApoE fractions from systemic background noise, (2) validating precise mathematical models that adjust peripheral biomarker concentrations against established indices of BBB permeability (such as the albumin quotient), and (3) characterizing the exact multi‐hour kinetic profile of ApoE production across diverse seizure etiologies. Without overcoming these vascular and logistical hurdles, dynamic ApoE monitoring will remain a retrospective indicator of neural injury rather than a predictive bedside guide for clinical management.

Finally, substantial clinical barriers remain before ApoE monitoring can be translated into bedside applications. Routine CSF sampling is impractical in most acute seizure settings, and plasma ApoE assays require rigorous standardization across laboratories, including control of genetic background, metabolic status, and peripheral lipid influences. Future development of rapid, minimally invasive assays combined with longitudinal biomarker profiling and multicenter validation will be essential to determine whether ApoE dynamics can serve as a reliable indicator of neural stress, recovery capacity, or long‐term neuropathological risk after seizures.

### 3.5 Clinical Heterogeneity and Patient Stratification

Emerging evidence suggests that the effects of ApoE4 are not uniform across all epilepsy populations. The magnitude and clinical manifestations of ApoE4‐associated risk appear to vary according to epilepsy subtype, age, ethnicity, disease duration, and the presence of neurodegenerative comorbidities. Current data indicate the strongest associations in mesial temporal lobe epilepsy, particularly among Han Chinese populations, whereas findings in other epilepsy syndromes remain inconsistent [29, 30, 32, 33]. Age‐dependent effects have also been observed, with older ApoE4 carriers exhibiting greater cognitive decline and seizure‐related memory impairment [28, 31]. Furthermore, prolonged disease duration may amplify ApoE4‐associated hippocampal injury and cognitive dysfunction [31]. Importantly, ApoE4 may interact with neurodegenerative processes, including tau accumulation and amyloid pathology. Therefore, epilepsy patients with Alzheimer‐related changes may represent a biologically distinct subgroup [8, 19, 42]. Collectively, these findings support a precision medicine framework in which ApoE4 is viewed not as a universal epilepsy risk factor but as a context‐dependent disease modifier whose impact varies across patient populations.

## 4 Therapeutic Strategies Targeting ApoE4‐Related Mechanisms in Epilepsy

**FIGURE 3:**
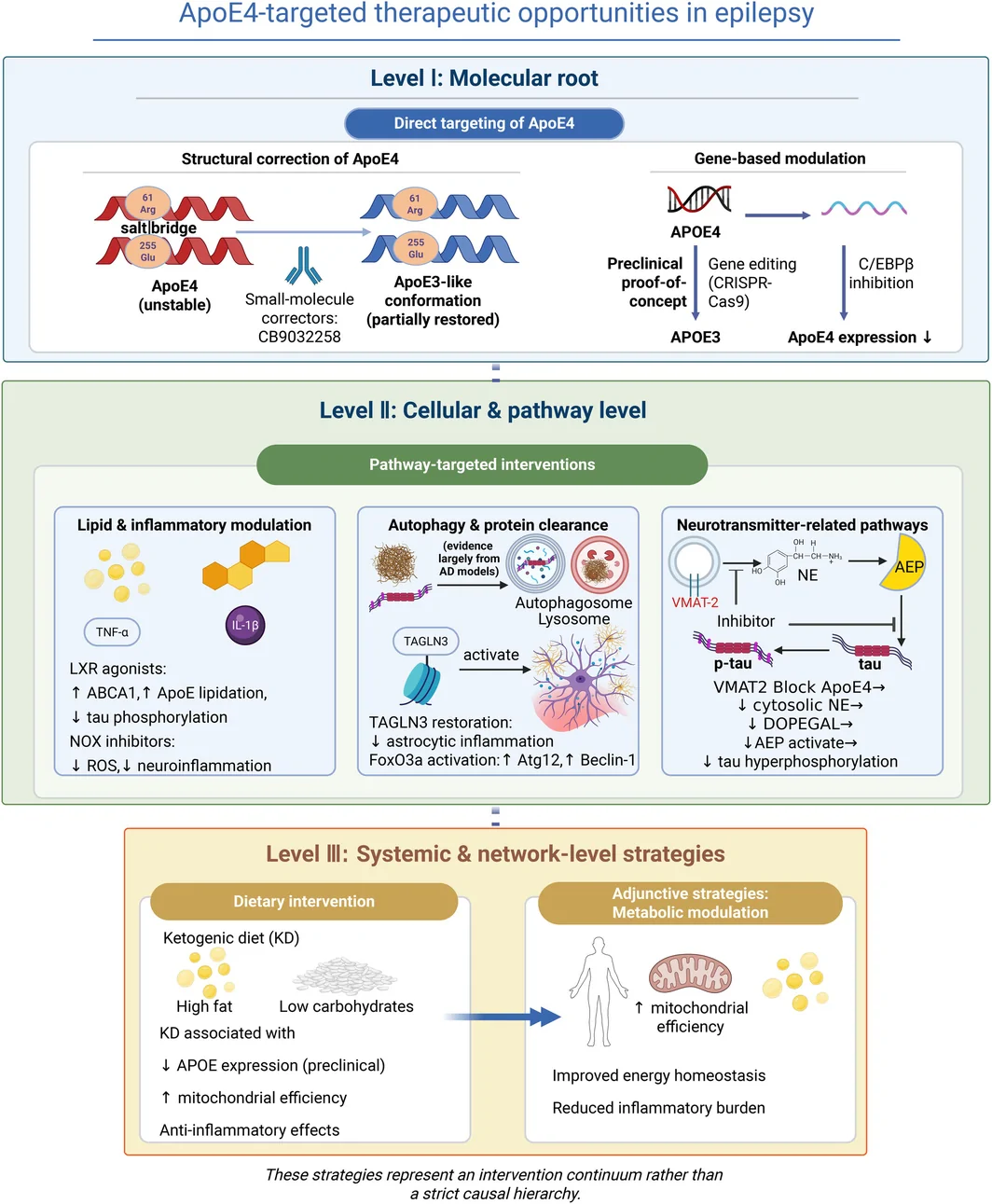
Therapeutic strategies targeting ApoE4 in epilepsy. Three‐level intervention gradient: (I) molecular root—direct targeting of ApoE4 through structural correction strategies (e.g., CB9032258) and gene‐based modulation approaches; (II) cellular/pathway level—targeting ApoE4‐associated downstream mechanisms, including lipid/inflammatory regulation, autophagy enhancement, and neurotransmitter‐related pathways. In particular, modulation of the ApoE4–VMAT2 axis may reduce cytosolic norepinephrine accumulation, limit DOPEGAL generation, and attenuate subsequent AEP activation and tau hyperphosphorylation‐associated pathology; (III) systemic/network level–ketogenic diet and whole‐body metabolic modulation strategies. These interventions represent a therapeutic continuum ranging from highly specific molecular approaches to broader network‐level strategies. AEP, asparagine endopeptidase; ApoE4, apolipoprotein E4; LXR, liver X receptor; NOX, NADPH oxidase; VMAT2, vesicular monoamine transporter 2. Created with http://biorender.com/.

Accumulating mechanistic insights position ApoE4 as a potential therapeutic target in epilepsy, thereby outlining conceptual opportunities for future targeted interventions that extend beyond conventional seizure suppression. Therapeutic strategies can be conceptually organized into three hierarchical levels (Figure 3): (i) Direct modulation of ApoE4, (ii) targeting of downstream pathogenic pathways, and (iii) systemic‐level interventions aimed at mitigating the broader metabolic and inflammatory milieu influenced by ApoE4. Collectively, these strategies provide a comprehensive framework for precision‐driven medicine approaches in ApoE4‐associated epilepsies.

### 4.1 Direct Modulation of ApoE4

The strategies outlined in this section were developed and validated primarily in AD preclinical models. Their translational relevance to epilepsy is based on shared mechanistic pathways (neuroinflammation, synaptic loss, and metabolic dysfunction) rather than direct anti‐seizure efficacy.

The most direct strategy to counteract ApoE4‐associated pathology in epilepsy is to target its aberrant protein structure or excessive gene expression, thereby intervening at the primary molecular source of its pathogenic effects.

Structural correction represents one preclinically promising but clinically unvalidated approach. ApoE4's distinct conformation predisposes it to unstable domain interactions, impaired lipid transport, and the induction of downstream synaptic dysfunction [43, 44, 45]. In AD models, small‐molecule correctors such as CB9032258 can disrupt these abnormal intramolecular interactions, shifting ApoE4 toward a more stable, ApoE3‐like conformation [46]. This conformational rescue has been demonstrated to restore mitochondrial energy function and promote neurite outgrowth. These processes that are especially relevant in epilepsy, where recurrent seizures impose substantial metabolic demand and exacerbate mitochondrial dysfunction, yet direct validation in epilepsy models is currently lacking [47, 48]. In parallel, ApoE4‐specific monoclonal antibodies (e.g., 9D11) have been demonstrated to reduce tau phosphorylation, reverse ApoE4‐driven cognitive deficits, and suppress neuroinflammation in AD models; however, their direct anti‐seizure efficacy remains unestablished [49]. Although epilepsy‐specific data remain limited, these effects are mechanistically relevant to epileptic syndromes characterized by progressive cognitive decline and tau‐related pathology, suggesting potential neuroprotective benefits beyond seizure control, though direct anti‐seizure efficacy has not been demonstrated.

At the transcriptional level, the transcription factor CCAAT/enhancer binding protein beta has been identified as a selective enhancer of APOE4 expression [50]. Pharmacological or genetic inhibition of this factor may reduce ApoE4 synthesis at its source, thereby attenuating downstream inflammatory and neurotoxic signaling cascades. Similarly, antisense oligonucleotide (ASO) therapies can reduce ApoE4 protein levels by approximately 50% and have demonstrated efficacy in alleviating tau pathology, synaptic loss, and glial activation in preclinical studies [51]. While direct epilepsy data are still lacking, ASO‐based approaches represent a highly specific precision‐medicine strategy that could theoretically be explored in ApoE4‐associated epilepsies with severe inflammation and cognitive involvement, pending preclinical epilepsy validation. Beyond ApoE4 itself, multi‐target ASOs—for example, those directed against tetratricopeptide repeat domain 39B—have improved metabolic parameters and reduced inflammation in other disease models [52], suggesting potential synergistic therapeutic benefits if applied to epilepsy.

Collectively, direct ApoE4 modulation targets the molecular origin of ApoE4 toxicity and represents a rational, genotype‐driven disease‐modifying strategy in preclinical models; clinical application to epilepsy remains experimental and requires extensive validation.

### 4.2 Pathway‐Targeted Interventions

The pathway‐targeted interventions discussed below derive largely from AD and neurodegeneration research. We present them as rational extensions of ApoE4 biology to epilepsy, pending validation in epileptogenesis models.

Given that ApoE4 drives epileptogenic cascades largely through downstream lipid, inflammatory, and synaptic pathways, an alternative therapeutic strategy is to modulate the cellular and molecular processes it disrupts, such as lipid metabolism, inflammation, autophagy, and neurotransmitter systems.

Targeting lipid and inflammatory signaling is a key approach, as ApoE4 exacerbates neuroinflammation by impairing cholesterol transport [53], altering membrane lipid composition [54], and stimulating the release of pro‐inflammatory mediators [55]. Liver X receptor (LXR) agonists have emerged as pleiotropic regulators of lipid metabolism and inflammation. In animal models, LXR activation upregulates the cholesterol transporter (ATP‐binding cassette sub‐family A member 1), increases ApoE lipidation, reduces Aβ42 accumulation, decreases hippocampal tau phosphorylation, and restores presynaptic integrity [56]. Although primarily documented in AD, these mechanisms directly intersect with core features of epileptic pathology, including lipid dysregulation and chronic neuroinflammation [57]. Nevertheless, because these pathways have not been evaluated in classical chemoconvulsant or genetic epilepsy models, their therapeutic translation remains purely speculative and represents a critical knowledge gap. Similarly, inhibitors of NADPH oxidase, a major generator of reactive oxygen species, effectively reduce oxidative stress and dampen neuroinflammation in animal and human models, thereby addressing a critical driver of epileptogenesis [58]. Such dual modulation of lipid and inflammatory pathways may be particularly advantageous in ApoE4 carriers exhibiting metabolic and inflammatory vulnerability.

Restoring autophagy and protein clearance represents another critical axis. ApoE4 represses transgelin 3 (TAGLN3) via the HDAC‐TAGLN3 pathway, promoting pro‐inflammatory astrocytic activation [59], and suppresses forkhead box protein O3‐A (FoxO3a) signaling, thereby reducing the expression of autophagy‐related proteins such as autophagy‐related 12 and beclin‐1 [60]. In epilepsy, where impaired autophagy contributes to maladaptive network remodeling, interventions that restore TAGLN3 signaling or enhance FoxO3a‐mediated autophagy may ameliorate neurodegeneration and promote network stabilization.

Modulating neurotransmitter metabolism offers a novel target. A newly identified mechanism in AD models highlights ApoE4's ability to bind the vesicular monoamine transporter 2 (VMAT2), inhibiting norepinephrine uptake and leading to cytosolic accumulation of this neurotransmitter [61]. The oxidative conversion of norepinephrine generates the toxic metabolite 3,4‐dihydroxyphenylglycolaldehyde, which activates AEP, promoting tau hyperphosphorylation and neurodegeneration [7]. Given that noradrenergic signaling from the locus coeruleus and tau pathology both contribute to seizure progression and cognitive decline, pharmacological inhibitors of the VMAT2‐ApoE4 interaction or direct inhibition of AEP activity may yield dual benefits by controlling seizures and preserving cognitive function.

Collectively, these pathway‐targeted strategies address the downstream consequences of ApoE4—lipid imbalance, inflammation, impaired clearance, and neurotransmitter dysregulation—providing a conceptual multipronged therapeutic framework that may 1 day modulate epileptogenic processes, pending rigorous experimental validation.

### 4.3 Systemic and Metabolic Approaches

Beyond molecularly targeted interventions, systemic strategies aimed at restoring metabolic and inflammatory balance may indirectly attenuate ApoE4‐associated pathology and are often more readily translatable to clinical practice.

Dietary interventions, particularly the ketogenic diet (KD), characterized by high fat and low carbohydrate intake, have long been recognized as an effective therapy for drug‐resistant epilepsy [62]. Notably, animal studies indicate that KD can downregulate APOE expression [63], suggesting that part of its antiseizure efficacy may involve modulation of ApoE4‐related pathways. For ApoE4 carriers, KD may therefore provide dual mechanistic benefits by reducing seizure burden while simultaneously mitigating ApoE4‐driven metabolic and inflammatory stress, in addition to its known effects on mitochondrial bioenergetics and neurotransmitter balance.

More broadly, systemic interventions that improve lipid handling, reduce inflammatory load, and enhance mitochondrial efficiency may lower the cumulative neuronal stress imposed by ApoE4. Although less specific than gene‐ or pathway‐targeted therapies, such approaches are clinically feasible and may serve as valuable adjuncts to existing antiseizure regimens, particularly in patients with metabolic vulnerability. Current and emerging therapeutic strategies targeting ApoE4‐related pathways are systematically categorized, including their mechanisms of action, developmental phases, and potential clinical indications (Table 3).

**TABLE 3:** ApoE4 targeted therapy strategy classification.

Types of interventions	Specific methods	Mechanism of action	Verified phase	Potential indications
Structural modification	CB9032258	Structural stability repair	Preclinical study (AD models)	Epilepsy with metabolic disorders, cognitive decline
Antibody blocking	Anti‐ApoE4 monoclonal antibody (9D11)	Blocking ApoE4 receptor	Preclinical study (AD models)	Epilepsy with AD‐like pathology and obvious inflammation
Gene regulation	Inhibitory C/EBPβ transcription factor	Reduced ApoE4 transcription levels	Preclinical study (AD models)	Epileptic subtypes suitable for high ApoE4 expression
Lipid‐inflammatory pathway regulation	LXR agonist	Increase ApoE lipidation	Preclinical study (AD models)	Epilepsy with lipid metabolism disorder and chronic inflammation
Oxidative stress inhibition	NADPH oxidase inhibitor	Reduce ROS production and inhibit neuroinflammation	Preclinical study (multiple disease models)	Epilepsy with obvious oxidative stress
Autophagy recovery	TAGLN3 signal enhancers or FoxO3a activators	Restore autophagy and promote harmful substance removal	Preclinical study (AD models)	Epilepsy with protein aggregation pathology
Regulation of neurotransmitter metabolism	VMAT2‐ApoE4 interaction inhibitors or AEP inhibitors	Block norepinephrine toxic metabolism, inhibit tau phosphorylation	Preclinical study (AD models)	Epilepsy with cognitive decline and tau pathology
Systemic metabolic intervention	Ketogenic diet	Down‐regulation of APOE expression, improvement of mitochondrial function, anti‐inflammatory	Already used clinically (refractory epilepsy)	Drug‐refractory epilepsy, ApoE4 carriers
Multi‐target ASO	Antisense oligonucleotides	Improve metabolic parameters, reduce inflammation	Preclinical study (epilepsy model)	Epilepsy with metabolism‐inflammation dual disorder

*Note:* Candidate interventions are grouped according to the hierarchical level at which they intercept ApoE4‐driven pathology: (i) structural or transcriptional correction of ApoE4 itself, (ii) downstream pathway modulation (lipid–inflammation, oxidative stress, autophagy, neurotransmitter metabolism), (iii) systemic metabolic intervention. “Specific methods” list the small molecules, biologics, dietary schemes, or nucleic‐acid therapeutics evaluated to date. “Mechanism of action” provides a concise mechanistic rationale linking the intervention to ApoE4 biology. “Verified phase” indicates the most advanced stage of validation in any neurological indication (preclinical or clinical). “Potential indications” suggests epilepsy subpopulations most likely to benefit, based on convergent biomarker or comorbidity profiles.

Abbreviations: AEP, asparagine endopeptidase; ASO, antisense oligonucleotide; FoxO3a, forkhead box protein O3‐A; LXR, liver X receptor; NADPH, reduced nicotinamide adenine dinucleotide phosphate; TAGLN3, transgelin‐3; VMAT2, vesicular monoamine transporter 2.

## 5 Discussions and Future Directions

### 5.1 Mechanistic Insights: From Shared Pathology to Epilepsy‐Specific Biology

Although accumulating evidence links ApoE4 to epilepsy through mechanisms that partially overlap with those observed in AD, whether ApoE4 acts merely as a shared risk factor or as an epilepsy‐specific disease modifier remains incompletely defined. Importantly, distinguishing these two possibilities is clinically relevant, because ApoE4 may represent either a primary modulator of epileptogenic network vulnerability or a genetic amplifier that accelerates seizure occurrence in the setting of latent neurodegenerative pathology. Current models implicate ApoE4 in epileptogenesis through neuroinflammation, impaired synaptic plasticity, metabolic dysregulation, and BBB dysfunction. Several of these mechanisms—including tau hyperphosphorylation [64, 65], aberrant GSK‐3β activation [66], and lipid imbalance [67]—are well established in AD and may contribute to epilepsy–dementia comorbidity. However, the extent to which these pathways independently drive seizure susceptibility, network instability, and cognitive decline in epilepsy remains to be determined through direct experimental validation.

To properly stratify the pathophysiological role of ApoE4, a critical mechanistic dichotomy must be considered: whether ApoE4 acts as an autonomous driver of epileptogenic network dysfunction or merely lowers seizure threshold secondary to ongoing neurodegenerative pathology. Current evidence increasingly supports the hypothesis that ApoE4 can independently promote epileptogenic network dysfunction. In human APOE targeted replacement mice, young ApoE4 knock‐in animals develop spontaneous hippocampal hyperexcitability. This phenotype is associated with increased interictal spike activity and E/I imbalance in the dentate gyrus and CA3 regions. Notably, these abnormalities occur before detectable amyloid pathology or cognitive impairment. Importantly, these abnormalities occur in the absence of familial AD mutations, indicating that ApoE4 itself is sufficient to induce pathological network instability. Mechanistically, ApoE4 promotes neuronal atrophy and intrinsic hyperexcitability in specific hippocampal neuronal populations, establishing a direct link between ApoE4 expression and epileptiform circuit dysfunction independent of classical AD pathology [68].

However, ApoE4 also functions as a potent disease modifier that may amplify neurodegenerative cascades once network instability is established. ApoE4‐mediated impairment of hilar GABAergic interneurons has been shown to be age‐dependent and Tau‐dependent, suggesting that Tau pathology progressively exacerbates inhibitory circuit failure [69]. In parallel, aged ApoE4‐KI mice exhibit disrupted sharp‐wave ripple‐associated slow gamma oscillations, a network phenotype that can be rescued by selective removal of ApoE4 from GABAergic interneurons, highlighting the central role of inhibitory dysfunction in linking epileptiform activity to cognitive decline. Together, these findings support a two‐stage model in which ApoE4 initially acts as a primary disruptor of hippocampal E/I balance and network excitability, thereby generating an epilepsy‐prone state independent of overt neurodegeneration [42]. Subsequently, Tau‐dependent interneuron dysfunction and other neurodegenerative processes further reinforce this instability, creating a feed‐forward cycle that couples epileptiform activity with progressive cognitive impairment. Therefore, ApoE4‐associated epilepsy should not be viewed solely as a secondary manifestation of AD but may, at least in a subset of individuals, represent an independent pathological process that precedes and potentially contributes to later neurodegeneration.

Building on this framework, pharmacological probes validated in AD—such as AEP inhibitors or modulators of VMAT2 function—could serve as mechanistic tools rather than therapeutic shortcuts, enabling rigorous testing of pathway relevance in ApoE4‐associated epilepsies. Furthermore, integrative multi‐omics approaches (including transcriptomics, lipidomics, and proteomics) applied to epilepsy models and patient samples may uncover context‐specific, ApoE4‐sensitive signaling networks that are seizure‐modulated, thereby refining therapeutic targeting. Clarifying the epilepsy‐specific actions of ApoE4 is therefore not merely an academic exercise but a critical prerequisite for rational, mechanism‐based therapeutic development.

### 5.2 Translational Barriers

Despite compelling preclinical rationale, the translation of ApoE4‐targeted strategies into epilepsy care faces substantial obstacles. A primary challenge remains the efficient and safe delivery of therapeutics across BBB, as many small molecules, biologics, and nucleic acid‐based therapies fail to achieve therapeutically meaningful concentrations in the central nervous system. Equally critical is isoform specificity. Because ApoE2 and ApoE3 exert essential physiological functions, therapeutic interventions must selectively modulate ApoE4 activity without disrupting normal lipid transport and synaptic maintenance mediated by other isoforms [70]. Although advanced drug delivery platforms, including nanocarriers and liposomal systems, have demonstrated promise in enhancing brain‐targeting capacity, significant concerns persist regarding their long‐term safety, physicochemical stability, and immunogenicity [71]. From a clinical standpoint, precision‐medicine approaches based on ApoE4 genotyping in epilepsy remain at an early stage of development. Progress will require large, ethnically diverse cohorts, standardized genotyping, longitudinal phenotyping, and multicenter collaboration to define which epilepsy subtypes, disease stages, and comorbidity profiles derive the greatest benefit from ApoE4‐targeted interventions. Addressing these translational barriers will therefore depend not only on technological advances but also on robust trial design, biomarker integration, and stratified patient selection strategies.

### 5.3 Multidisciplinary Innovation

Advancing the field of ApoE4 in epilepsy will necessitate multidisciplinary innovation, leveraging cutting‐edge models and technologies for mechanistic discovery and therapeutic design. Brain organoid models, combined with single‐cell transcriptomics, offer powerful platforms to decode ApoE4 dynamics across neuronal and glial subtypes during epileptogenesis [72]. Gene‐editing technologies such as CRISPR/Cas9 have already corrected APOE4 to APOE3 in preclinical systems [73], reducing tau phosphorylation and improving neuronal function. These approaches provide unprecedented opportunities for mechanistic dissection and therapeutic innovation, but challenges in delivery efficiency, off‐target effects, and long‐term safety must be addressed before clinical application.

## 6 Conclusion and Future Perspectives

ApoE4 emerges from current evidence as a context‐dependent disease modifier in epilepsy, influencing seizure susceptibility, disease progression, cognitive outcomes, and treatment responsiveness. By amplifying neuroinflammation, impairing synaptic plasticity, disrupting lipid and energy metabolism, compromising BBB integrity, and promoting tau‐related pathology, ApoE4 reconfigures epileptic networks across molecular, cellular, and systems levels. Consequently, these same mechanisms create actionable entry points for targeted intervention, positioning ApoE4 at the critical interface between pathogenesis and therapeutic innovation.

Epilepsy is a multifactorial disorder shaped by genetic background, environmental exposures, and network‐level pathology. The contribution of ApoE4 likely spans these levels, necessitating an integrated framework that bridges genetics, immunology, metabolism, and systems neuroscience. Future progress in this field should prioritize three interconnected goals: (i) Defining epilepsy‐specific ApoE4 signaling pathways and distinguishing them from shared neurodegenerative mechanisms; (ii) developing isoform‐selective, BBB‐permeable interventions with demonstrable disease‐modifying potential in experimental models; (iii) integrating multidisciplinary innovation with well‐designed early‐phase clinical studies to translate mechanistic insights into therapeutic strategies.

Although ApoE4‐targeted therapies in epilepsy remain at an exploratory stage, rapid advances in experimental modeling, molecular targeting, and precision medicine provide a credible and achievable translational pathway forward. With rigorous mechanistic validation and strategic clinical translation, ApoE4‐focused approaches may ultimately expand treatment options for selected epilepsy subgroups and redefine the role of genetic modifiers in the precision management of epilepsy.

## Author Contributions

K.P. and S.D. drafted and revised the manuscript. S.D. and Y.H. conceived and designed the review. K.P. and K.Z. performed literature searches. Y.E. revised the manuscript. All authors read and approved the final manuscript.

## Funding

This study was supported by the Zhejiang Provincial Natural Science Foundation (LJHSQY26H090003) and the Major (Key) Science and Technology Project of Jinhua City (grant no. 2026‐3‐127).

## Ethics Statement

The authors have nothing to report.

## Consent

The authors have nothing to report.

## Conflicts of Interest

The authors declare no conflicts of interest.

## Data Availability

The authors have nothing to report.
